# LncRNA MAGI2-AS3 Overexpression Sensitizes Esophageal Cancer Cells to Irradiation Through Down-Regulation of HOXB7 *via* EZH2

**DOI:** 10.3389/fcell.2020.552822

**Published:** 2020-11-24

**Authors:** Wenfang Cheng, Xiuling Shi, Mingqiang Lin, Qiwei Yao, Jiayu Ma, Jiancheng Li

**Affiliations:** Department of Radiation Oncology, Fujian Medical University Cancer Hospital, Fujian Cancer Hospital, Fuzhou, China

**Keywords:** long non-coding RNA MAGI2-AS3, homeobox protein Hox-B7, esophageal cancer, radio-resistance, enhancer of zeste homolog 2, trimethylation of lysine 27 on histone H3

## Abstract

**Background:**

Accumulating evidence has suggested that aberrant expression of long non-coding RNAs (lncRNAs) may contribute to cancer progression in association with radioresistance. The current study aimed to identify the potential role of lncRNA MAGI2-AS3 and the underlying mechanism in its regulation of the radio-sensitivity of esophageal cancer cells.

**Methods and Results:**

Initially, we detected high expression of HOXB7 from microarray-based gene expression profiling of esophageal cancer. Then, we identified the interactions among MAGI2-AS3, HOXB7, and EZH2 by dual-luciferase reporter gene assay, RNA pull-down assay, RIP assay and ChIP assay. HOXB7 was highly-expressed, while MAGI2-AS3 was poorly-expressed in esophageal cancer tissues and cells. The effect of MAGI2-AS3 and HOXB7 on esophageal cancer cell proliferation and apoptosis as well as tumorigenicity of radioresistant cells was examined by gain- and loss-of-function experiments. Interestingly, MAGI2-AS3 down-regulated HOXB7 through interaction with EZH2, which promoted cell apoptosis and inhibited proliferation and radio-resistance. Besides, down-regulation of MAGI2-AS3 exerted a promoting effect on these malignant phenotypes.

**Conclusion:**

Taken together, our results reveal the potential role of MAGI2-AS3 over-expression in controlling esophageal cancer resistance to radiotherapy by down-regulating HOXB7, this providing a candidate biomarker for resistance to radiotherapy.

## Introduction

Esophageal cancer is one of the most aggressive gastrointestinal cancers and one of the leading causes of cancer-related deaths across the world, placing a substantial burden on the quality of life among patients undergoing treatment (Lagergren et al., 2017). The primary histological subtypes of esophageal cancer, adenocarcinoma and squamous cell carcinoma, arise from different causes (Lagergren and Lagergren, 2010). Current mainstay treatment modalities include targeted therapies, immunotherapies, resection, chemoradiotherapy, and preoperative chemotherapy (Hamamoto et al., 2018; Barsouk et al., 2019). Although these therapies have significantly improved the survival for early stage patients, a large proportion of patients plagued by metastatic esophageal cancer still exhibit poor survival rates (Barsouk et al., 2019). Fortunately, accumulating evidence has indicated that primary tumor directed radiotherapy is associated with the overall survival improvement in patients with newly diagnosed metastatic esophageal cancer (Guttmann et al., 2017). Advancements in radiotherapy in the context of esophageal cancer have also been widely-documented (Xi and Lin, 2017).

It is worth noting that radiotherapy resistance of cancers still remains a major obstacle for efficient treatment of cancers, although long non-coding RNAs (lncRNAs) have been implicated in the regulation of radiotherapy resistance (Chi et al., 2017). For instance, lncRNA-p21 was indicated to promote the radiotherapy sensitivity of gastric cancer (Chen et al., 2019). Besides, lncRNA FAM201A has also been uncovered as a potential biomarker for radio-resistance in esophageal squamous cell carcinoma (ESCC) (Chen et al., 2018). Microarray-based analyses in the current study identified another differentially expressed lncRNA, MAGI2-AS3, along with a differentially expressed gene (DEG), homeobox protein Hox-B7 (HOXB7) in esophageal cancer. To the best of our knowledge, only two previous studies have explored the role of MAGI2-AS3 in cancers. One functional study highlighted MAGI2-AS3 as a bladder cancer suppressor (Wang et al., 2018), while the other study focused on its effects on breast cancer cell growth (Yang et al., 2018). A member of HOX family, HOXB7 has also been highlighted to participate in a variety of crucial cellular processes during oncogenesis (Errico et al., 2016), with particular involvement in gastric cancer, wherein its over-expression promoted cell migration and invasion while restraining cell apoptosis (Joo et al., 2016). More importantly, over-expression of HOXB7 imparts reduced sensitivity of oral cancer cells to chemo-radiotherapy (Yuan et al., 2016). Based on the aforementioned literature review, we hypothesized that MAGI2-AS3 and HOXB7 might together play specific roles in the occurrence and development of esophageal cancer, especially from the perspective of radiotherapy sensitivity. Therefore, we designed the current study to test this hypothesis, aiming to provide useful molecular markers to predict the prognosis and guide the treatment of esophageal cancer.

## Materials and Methods

### Ethics Statement

The study was conducted under the approval of the Institutional Review Board of Fujian Medical University Cancer Hospital, Fujian Cancer Hospital and performed in strict accordance with the *Declaration of Helsinki*. Signed informed consents were obtained from all participants prior to enrollment. All animal experiments were performed in accordance with the Guide for the Care and Use of Laboratory animals published by the United States National Institutes of Health. Extensive efforts were made to ensure minimal suffering of the included animals.

### Microarray-Based Gene Expression Profiling

The esophageal cancer-related microarray data GSE45670 and the annotated probe files were retrieved from the Gene Expression Omnibus (GEO)^1^. The Affy installation package of R software was applied to normalize the microarray data (Gautier et al., 2004) and the Limma package was used to screen the DEGs (Smyth, 2004) with | log2FoldChange| > 2.0 and *p*-value < 0.05 serving as the threshold. Subsequently, a heat map of the obtained DEGs was plotted. In addition, the expression levels of DEGs were verified by Gene Expression Profiling Interactive Analysis (GEPIA)^2^, where the data were retrieved from The Cancer Genome Atlas (TCGA) and Genotype-Tissue Expression (GTEx) databases (Tang et al., 2017). The correlation between DEG expression and MAGI2-AS3 expression was subsequently analyzed.

### Patient Enrollment

A total of 92 cases of surgically resected specimens (66 males and 26 females, aged 33–71 years, with a mean age of 56 years) pathologically confirmed as esophageal cancer from January 2015 to January 2018 at the Department of Thoracic Surgery of Fujian Medical University Cancer Hospital, Fujian Cancer Hospital were included in the current study. Additionally, adjacent normal tissues (3–5 cm from the carcinomas) were collected as control material. None of the included patients had undergone anti-cancer therapies prior to their entry in the study. The resected tumors were categorized according to the World Health Organization (WHO) classification (Yanagawa et al., 2012) in combination with morphological observation as follows: well differentiated (*n* = 25); moderate differentiation (*n* = 45); poor differentiation (*n* = 22). According to the tumor-nodes-metastasis (TNM) classification (Nomura et al., 2012), the included specimens were stage I + II (*n* = 45); stage III + IV (*n* = 47). A portion of the tissues was stored at −80°C and the other portion was fixed in 10% formaldehyde, paraffin-embedded and sliced at a thickness of 4 μm.

### Immunohistochemistry (IHC)

The positive expression of HOXB7 protein was detected using the immunohistochemical streptavidin peroxidase (SP) method. In brief, the aforementioned paraffin-embedded specimens were sliced at a thickness of 4 μm, and subjected to conventional gradient alcohol dehydration. The sections were then blocked with 3% H_2_O_2_ for 10 min at room temperature to eliminate endogenous peroxidase activity, followed by another 10 min of blocking with the addition of normal non-immune animal serum. Next, the samples were incubated with the primary rabbit polyclonal antibody to HOXB7 (ab196007, dilution ratio of 1:100, Abcam Inc., Cambridge, United Kingdom) at 4°C overnight, then incubated with the biotin-labeled secondary goat anti-rabbit immunoglobulin G (IgG) (dilution ratio of 1:500) at 37°C for 20 min and finally incubated with 50 μL streptavidin-biotin-peroxidase at room temperature for 10 min. Subsequently, diaminobenzidine tetrahydrochloride (DAB) was added to the sections for coloration, followed by hematoxylin counterstaining, dehydration, clearing, mounting and observation under a microscope. The primary antibody was replaced by phosphate buffer saline (PBS) as the negative control (NC). Five fields of view were randomly selected, followed by cell counting. Two pathologists blind to the clinical data assessed IHC staining scores of HOXB7 in liver tissues using a semi-quantitative method. The staining scores were assessed as 0 (negative), 1 (weak), 2 (moderate), and 3 (strong). High expression was defined as a staining score of ≥2 with at least 50% of malignant cells showing positive HOXB7 staining, and low expression was defined as <50% of malignant cells showing nuclear staining or a staining score <2 (Huan et al., 2017).

### Cell Culture

Normal esophageal epithelial cell line (HEEC) and three esophageal cancer cell lines (KYSE30, KYSE150 and KYSE450) were purchased from The Institute of Hematology & Oncology of Heilongjiang Province (Harbin, Heilongjiang, China). The esophageal cancer cells were removed from the cryopreservation box and immediately placed in a warm bath at 37°C for cell recovery. The well-recovered cells were added to a centrifuge tube, followed by the addition of calf serum-free Roswell Park Memorial Institute (RPMI) 1640 medium (Santa Cruz Biotechnology Inc., Santa Cruz, CA, United States) for cell re-suspension. After centrifugation at 1500 rpm for 5 min the supernatant was discarded and the cell pellet was suspended in 15% fetal bovine serum (FBS) and incubated at 37°C with 5% CO_2_ in air. The expression of HOXB7 was then determined using the reverse transcription quantitative polymerase chain reaction (RT-qPCR), and the cell line exhibiting the highest HOXB7 expression was selected for subsequent experimentation. The complementary DNA (cDNA) full-length sequences (Ensembl Genome Browser) of MAGI2-AS3 and HOXB7, along with corresponding NC sequences, were designed using software purchased from the ABI Company (Oyster Bay, NY, United States). LNA-GapmeR technology was employed to interfere with MAGI2-AS3 expression by short hairpin RNA (sh)-MAGI2-AS3. Construction of lentivirus interference vectors and shRNA vectors, sequencing, virus package, and titer determination were entrusted to GeneChem Co., Ltd. (Shanghai, China).

### Construction of Radioresistant Cell Lines

Esophageal cancer cells at the logarithmic phase of growth were exposed to linac X-ray radiation (X-ray tube voltage: 150 KV, X-ray tube current: 20 mA, air kerma rate at the probe: 7.18 Gy/min, air kerma rate at the object: 4.81 Gy/min). The distance between focus and object was set as 350 nm, and the initial dose was 1 Gy. The medium was renewed after every exposure to X-rays. When cell confluence reached 90%, the cells were passaged and plated. Exposure at a dose of 1 Gy was repeated when the cells entered the logarithmic growth phase. The dose was gradually increased (1 Gy for 3 times, 2 Gy for 3 times, and 4 Gy for 7 times). The esophageal cancer cell line with highest radio-resistance was identified as KYSE150R. The obtained cells were used immediately for experimentation or stored in liquid nitrogen. The parental cells were treated under the same conditions except that they were spared irradiation. The total number of surviving cells was determined using the trypan blue dye exclusion test, where survival rate of the cell line = the number of viable cells in the cell line/total number of viable cells in the untreated group ×100% (Jin et al., 2018).

### Cell Treatment

The esophageal cancer cells at the logarithmic phase of growth were plated in a 24-well plate. When reaching approximately 50% confluence, the KYSE150 and KYSE150R cells were infected with lentiviral vectors carrying overexpression (oe)-MAGI2-AS, sh-MAGI2-AS3, oe-HOXB7 or the corresponding NC sequences (oe-M-NC, sh-M-NC, sh-NC and oe-NC), respectively. sh-MAGI2-AS3: 5′-gtgagacattaacagtgatttaa-3′; sh-HOXB7: 5′-atgcgaatactttattttctaaa-3′. The medium was renewed after 24 h of culture. Following additional 48 h of incubation at 37°C with 5% CO_2_ in air, the cells in stable passage were screened for subsequent experimentation. Lentiviral vectors carrying oe-M-NC, sh-M-NC, sh-NC, sh-MAGI2-AS3 (Pu et al., 2019), sh-HOXB7 (Huo et al., 2019) and oe-NC were purchased from Shanghai GenePharma Co., Ltd. (Shanghai, China). Each reaction was performed in triplicate.

### RNA Isolation and Quantitation

Total RNA content was extracted from tissues or cells using TRIzol reagents (15596-018, Solarbio Life Sciences Co., Ltd., Beijing, China) according to the instructions. Subsequently, the concentration of the obtained RNA was determined. The primers were synthesized by Takara Co., Ltd. (Dalian, Liaoning, China) (Table 1).

**TABLE 1:** Primer sequences for reverse transcription quantitative polymerase chain reaction.

Gene	Forward sequence	Reverse sequence
MAGI2-AS3	5′-CCAGTGCGGACCTTTCTTCA-3′	5′-CTCTTGGATGCAAACGGCAG-3′
HOXB7	5′-TATGGGCTCGAGCCGAGTT-3′	5′-GGCCTCGTTTGCGGTCAGT-3′
GAPDH	5′-TGAACGGGAAGCTCACTGG-3′	5′-TCCACCACCCTGTTGCTGTA-3′

*HOXB7, homeobox protein Hox-B7; GAPDH, glyceraldehyde-3-phosphate dehydrogenase.*

Reverse transcription was conducted following the protocols of the cDNA RT kit (K1622, Reanta Biotechnology Co., Ltd., Beijing, China). Quantification was performed using the fluorescent qPCR instrument (ViiA 7, Daan Gene Co., Ltd., Guangzhou, Guangdong, China). With glyceraldehyde-3-phosphate dehydrogenase (GAPDH) serving as the internal control, the fold change of gene expression was calculated using the 2^–ΔΔ*Ct*^ method (Ayuk et al., 2016). The experiment was performed in triplicate.

### Western Blot Analysis

Total protein content was extracted from tissues or cells using high-efficiency radioimmunoprecipitation assay (RIPA) lysis buffer (R0010, Solarbio Life Sciences Co., Ltd., Beijing, China). The protein concentration was then determined using a bicinchoninic acid (BCA) kit (20201ES76, Yeasen Biotechnology Co., Ltd., Shanghai, China). The obtained proteins were separated by sodium dodecyl sulfate-polyacrylamide gel electrophoresis and transferred onto a polyvinylidene fluoride membrane using the wet-transfer method. The membrane was blocked with 5% bovine serum albumin (BSA) at room temperature for 1 h and then incubated at 4°C overnight with diluted primary rabbit antibodies to HOXB7 (ab196007, dilution ratio of 1:1000), Ki67 (ab92742, 1: 5000), proliferating cell nuclear antigen (PCNA) (ab92552, 1: 1000), Cyclin D1 (ab16663, 1: 200), CDK4 (ab68266, 1: 1000), caspase 3 (ab13847, 1: 500), Bcl-2-associated X protein (Bax) (ab18283, 1: 1000), B-cell lymphoma 2 (Bcl-2) (ab59348, 1: 500), and GAPDH (ab181602, 1:10,000). The membrane was then incubated with the horseradish peroxidase (HRP)-conjugated secondary goat anti-rabbit antibody to IgG (ab205718, dilution ratio of 1:20,000) at room temperature for 1 h, followed by 3 washes with Tris-buffered saline Tween-20 (TBST). All aforementioned antibodies were purchased from Abcam Inc. (Cambridge, United Kingdom). The membrane was then developed. The protein bands were photographed and the gray value was quantified and analyzed using the ImageJ 1.48u software (National Institutes of Health, Bethesda, MD, United States). GAPDH was used as the internal control, and the relative expression of each protein was expressed as the ratio of the gray value of each protein to GAPDH. The experiment was performed in triplicate.

### Colony Formation Assay

KYSE150 and KYSE150R cells were plated in 6-well plates. The cells exposed to 0 Gy were seeded at a density of 500 cells/well, 2 Gy at 1000 cells/well, 4 Gy at 2000 cells/well, 6 Gy at 3000 cells/well, and 8 Gy at 4000 cells/well. Triplicate wells were set for each group. After adhering to the wells, the cells were exposed to X-ray at doses of 0, 2, 4, 6, or 8 lasting for 10 days. The experiment was terminated when the cell clones were visible to the naked eye, whereupon 50 cells in each clone were observed under a microscope. Following PBS rinsing, the cells were fixed with 500 μL of 4% paraformaldehyde for 15 min, stained with 500 μL of crystal violet for 15 min, washed, dehydrated, cleared, and photographed. The number of clones with more than 50 cells was then counted. Plating efficiency (PE) = the number of clones/the number of plated cells × 100%, and survival fraction (SF) = PE _*the experimental group*_/PE _*the control group*_ × 100%. The experiment was performed in triplicate.

### Fluorescence *in situ* Hybridization (FISH) Assay

Information regarding the sub-cellular localization of MAGI2-AS3 was retrieved online^3^, which was then verified by FISH following the instructions of the RiboTM lncRNA FISH probe Mix (Red) (Ribo Biotechnology Co., Ltd., Guangzhou, Guangdong, China). The MAGI2-AS3 probe sequence used in the FISH assay was CATTACAGCTCGGCTACTGC. A coverslip was placed in a 6-well plate, where the cells were seeded in a 24-well plate. When the cell confluence reached about 80%, the coverslip was rinsed with PBS, fixed with 1 mL of 4% paraformaldehyde, and treated with proteinase K (2 μg/mL), glycine and the acetylation reagent. Then, the cells were prehybridized with 250 μL of prehybridization solution at 42°C for 1 h. After removing the prehybridization solution, 250 μL of hybridization solution (300 ng/mL) containing the probe was added for hybridization at 42°C overnight. After 3 rinses with phosphate buffered saline with Tween-20 (PBST), the nucleus was stained with PBST-diluted 4′,6-diamidino-2-phenylindole (DAPI) (dilution ratio of 1:800) for 5 min, followed by 3 PBST rinses. Five visual fields were randomly selected and photographed under a fluorescence microscope (Olympus Corp., Tokyo, Japan). The experiment was performed in triplicate.

### Dual-Luciferase Reporter Gene Assay

The binding between the promoter region of HOXB7 and MAGI2-AS3 was predicted by an online Blast website^4^. Subsequently, the target relationship between HOXB7 and MAGI2-AS3 was verified using dual-luciferase reporter gene assay. According to the binding sequence between the promoter region of HOXB7 and MAGI2-AS3, luciferase reporter gene vectors of pGL3-HOXB7 wild type (WT) (GGAGGGGAGCCAAG) and pGL3-HOXB7 mutant (MUT) (AACAAAACAAACCA) (pGL3-Promoter, HZ0194, Shanghai and Shanghai Zhen Biotechnology Co., Ltd., Shanghai, China) were constructed as previously described (Cai et al., 2019). The two plasmids were subsequently co-infected with MAGI2-AS3 over-expressing vector or the NC plasmid into the KYSE150 cells. The cells were lysed after 48 h. The supernatant was obtained through centrifugation at 12,000 rpm for 1 min. Luciferase activity was measured using a Dual-Luciferase^®^ Reporter Assay System (E1910, Promega Corp., Madison, WI, United States) with Renilla luciferase serving as the internal control. The relative luciferase activity = Firefly luciferase activity/Renilla luciferase activity.

### RNA Pull-Down Assay

T7 RNA polymerase (Ambion, United States) was used to transcribe the MAGI2-AS3 fragment *in vitro*, which were then treated with RNeasy Plus Mini kit (Qiagen, Germany), DNase I (Qiagen, Germany), and purified by RNeasy Mini Kit. The 3′end of the purified RNA was biotinylated with a biotin RNA labeling mixture (Ambion, United States). Next, 1 μg of labeled RNA was heated to 95°C in an RNA structure buffer (10 mM Tris pH7, 100 mM KCl, and 10 mM MgCl_2_). After 2 min, the sample was transferred to ice and incubated for 3 min. The mixture was let to stand for 30 min to allow the RNA to form a suitable secondary structure. Then, 3 μg of cells was subjected to lysis buffer (Sigma, United States) at 4°C for 1 h. The lysate was centrifuged at 12,000 × *g* at 4°C for 10 min, and the supernatant was collected and transferred to a RNase-free centrifuge tube. Biotinylated RNA (400 ng) was added with 500 μL RIP buffer and mixed with cell lysate, followed by incubation for 1 h at room temperature. The streptavidin magnetic beads was added to each binding reaction and incubated at room temperature for 1 h. After five washes with RIP buffer, 5× loading buffer was added to incubate the mixture at 95°C for 5 min. Finally, Western blot assay was performed to measure the eluted enhancer of zeste homolog 2 (EZH2) protein.

### RNA Binding Protein Immunoprecipitation (RIP) Assay

RNA binding protein immunoprecipitation assay was performed according to the instructions of the Magna RIP RNA-Binding Protein Immunoprecipitation kit (Millipore, Temecula, CA, United States). The cells at the logarithmic phase of growth were rinsed with pre-cooled PBS twice and lysed with 100 μL of lysis buffer containing protease inhibitor and ribonuclease inhibitor on ice for 30 min, followed by centrifugation at 12000 rpm and 4°C for 3 min. A portion of the supernatant was used as Input for positive control. Corresponding antibodies (1 μg), including normal mouse NC antibody to IgG and target protein specific rabbit antibody to EZH2 (ab195409, Abcam Inc., Cambridge, MA, United States), and 10–50 μL of protein A/G-beads were mixed with the remaining supernatant. Following incubation at 4°C overnight, the supernatant was removed by centrifugation at 3000 rpm for 5 min at 4°C. The protein A/G-beads were washed with 1 mL of lysis buffer for 3–4 times. After addition of 15 μL of 2× sodium dodecyl sulfate, the sample was heated in boiling water for 10 min. RNA content was isolated from the precipitate and purified using the conventional TRIzol method. The binding between MAGI2-AS3 and EZH2 was verified (Table 1). The experiment was performed in triplicate.

### Chromatin Immunoprecipitation (ChIP) Assay

Enrichment of EZH2 in the promoter region of HOXB7 was evaluated using a ChIP kit (Millipore, Temecula, CA, United States). The cells at the logarithmic phase of growth were fixed with 1% formaldehyde for 10 min upon reaching 70–80% confluence to initiate DNA-protein crosslinking. The chromatin fragments were obtained by sonication (10 s each time at an interval of 10 s, 15 times in total). The supernatant was collected by centrifugation at 13,000 rpm and 4°C, sub-packed into 3 tubes, and incubated with the normal mouse antibody to IgG or the target protein specific rabbit antibody to EZH2 (ab195409, Abcam Inc., Cambridge, United Kingdom) or trimethylated lysine-27 of histone H3 (H3K27me3) (ab192985, Abcam Inc., Cambridge, United Kingdom) at 4°C overnight. The endogenous DNA-protein complex was precipitated using Protein Agarose/Sepharose. The precipitate was obtained and de-crosslinking was conducted at 65°C overnight and the obtained DNA fragments were purified and extracted by phenol/chloroform. The Input (partial DNA fragments) was regarded as the internal control. The ChIP primer sequences 5′-GTCCCTGCCTACAAATCATCC-3′ (forward) and 5′-GGAAGCAAACGCACAAGAAGT-3′ (reverse) were used for HOXB7 (Zhang et al., 2014). The binding of EZH2 to HOXB7 promoter region was detected in triplicate.

### 5-Ethynyl-2′-Deoxyuridine (EdU) Staining

The cells at the logarithmic phase of growth 48 h post infection were labeled by incubation in EdU medium for 2 h (100 μL/well). The cells were then incubated with glycine (2 mg/mL) for 5 min, rinsed with PBS for 5 min (100 μL/well) and then incubated with PBS containing 0.5% Triton X-100 (100 μL/well) for another 10 min. Afterward, the cells were stained with 1× Apollo liquor for 30 min and 1× Hoechst 33342 (100 μL/well) in subdued light. Six to ten fields of view were randomly selected for observation and photography under a fluorescence microscope. The number of EdU-labeled cells was counted and recorded. The cells with red-stained nucleus were regarded as positive cells. EdU labeling (%) = the number of positive cells/(the number of positive cells + the number of negative cells) × 100%. The experiment was performed in triplicate.

### Flow Cytometry

Cell cycle distribution was detected as follows. The cells were harvested and rinsed with cold PBS for 3 times. The cells were then re-suspended in PBS with the concentration adjusted to 1 × 10^5^ cells/mL. For each test, 1 × 10^5^ cells were used. The cells were fixed with 1 mL of 75% ethanol (pre-cooled at −20°C) at 4°C for 1 h, and rinsed twice with cold PBS. Then, 100 μL of RNase A was added and incubated with the cells at 37°C for 30 min, whereupon the cells were stained with 400 μL of propidium iodide (PI) (Sigma-Aldrich Chemical Company, St Louis, MO, United States) at 4°C in subdued light. The cell cycle was detected after recording the red fluorescence at an excitation wavelength of 488 nm using flow cytometry.

Cell apoptosis was measured with the same cell processing. Based on the instructions of the Annexin-V-fluorescein isothiocyanate (FITC) cell apoptosis detection kit (Sigma-Aldrich Chemical Company, St Louis, MO, United States), the Annexin-V-FITC/PI liquor was prepared by mixing Annexin-V-FITC, PI and 2-[4-(2-hydroxyethyl)-1-piperazinyl]ethanesulfonic acid (HEPES) at a ratio of 1:2:50. For each test, 1 × 10^6^ cells were resuspended with 100 μL of dye liquor and incubated at room temperature for 15 min. The cells were then washed and resuspended with 500 μL of HEPES. Cell apoptosis was analyzed through detection of FITC and PI fluorescence by activating the band pass at 525 and 620 nm by a wavelength of 488 nm using flow cytometry.

### Xenograft Tumor in Nude Mice

A total of 90 BALB/c nude mice of either sex (aged 4–8 weeks) were housed under constant temperature of 25–27°C and constant humidity of 45–50%. KYSE150R cells infected with sh-MAGI2-AS3, oe-MAGI2-AS3, oe-HOXB7 or oe-MAGI2-AS3 + oe-HOXB7 along with corresponding NC (sh-M-NC or oe-M-NC) were detached, rinsed 2–3 times with PBS, and formulated into a single cell suspension at a density of 1 × 10^7^ cells/mL. Twenty micro liter of cell suspension was implanted subcutaneously in the back of each mouse. When the volume of transplanted tumor reached 1000 mm^3^, the nude mice with tumor of equal volume were exposed to linac X ray at a dose of 0, 2, 4, 6, and 8 Gy, respectively. After 14 days, the mice were euthanized by anesthesia overdose and the tumors were resected, photographed, weighed, and measured. A growth curve was then drawn by plotting the tumor volume against time. The tumor volume was calculated according to the following formula: V (mm^3^) = (a × b^2^)/2 (a, long axis; b, short axis) (Kun-Peng et al., 2017). The experiment was performed in triplicate.

### Statistical Analysis

Statistical analyses were performed using the SPSS 21.0 software (IBM Corp., Armonk, NY, United States). The enumeration data were expressed as n (%) and analyzed using the chi-square test. Measurement data were expressed as mean ± standard deviation. Normal distribution and homogeneity of variance were performed for all data. Data conforming to normal distribution and homogeneity of variance between two groups were compared by independent sample *t*-test, while data between cancer and adjacent normal tissues were compared by paired *t*-test. Data among multiple groups were assessed by one-way analysis of variance or repeated measures analysis of variance. Pairwise comparison within one group was analyzed by the Tukey’s test. The rank-sum test was applied for samples with defect variances. All comparisons were conducted by two-tailed test. A value of *p* < 0.05 was indicative of statistical significance.

## Results

### HOXB7 Is Highly Expressed in Esophageal Cancer

**FIGURE 1:**
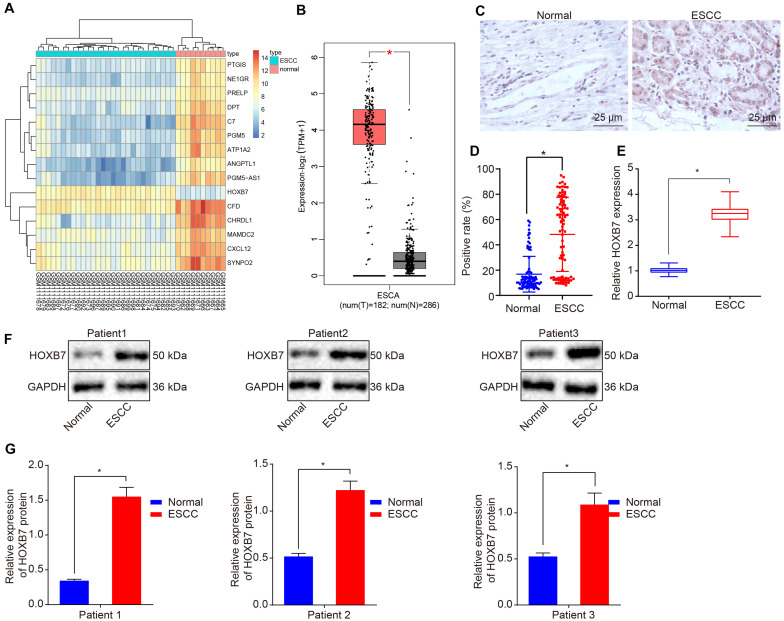
High expression of HOXB7 in esophageal cancer. **(A)** Differentially expressed genes related to esophageal cancer in the GSE45670 dataset; the abscissa represents the sample number, the ordinate represents differentially expressed genes, the upper right histogram represents color gradation and each rectangle corresponds to the expression value. **(B)** Differential expression of HOXB7 in esophageal cancer collected by TCGA and GTEx databases. The abscissa represents the sample type, the ordinate represents the expression value; the red box diagram represents the tumor sample, and the gray box diagram represents the normal sample (*q* < 0.01). **(C)** IHC analysis of HOXB7 protein in adjacent normal and esophageal cancer tissues (*n* = 92) (400×). **(D)** Quantitation of panel **(C)**. The positive expression of HOXB7 protein was measured using immunohistochemical SP method following the sectioning of the paraffin specimens of esophageal cancer and adjacent normal tissues (*n* = 92). **(E)** mRNA expression of HOXB7 determined by RT-qPCR in adjacent normal and esophageal cancer tissues (*n* = 92). **(F)** Representative Western blots of HOXB7 protein in adjacent normal and esophageal cancer tissues (*n* = 92), normalized to GAPDH. **(G)** Quantitation of panel **(F)**. *p < 0.05 versus the adjacent normal tissues. The data in panel **(D)** were enumeration data, expressed as n (%) and analyzed by chi-square test. The other data were measurement data and expressed as mean ± SD. Comparison between two groups was conducted by paired *t*-test. The experiment was performed in triplicate.

We first performed a differential analysis on the esophageal cancer-related expression dataset GSE45670. A heat map illustrating the most DEGs is shown in Figure 1A, which depicts that HOXB7 was the most significantly highly differentially expressed gene in esophageal cancer. Then, the GEPIA2 database^5^ was used to retrieve the expression of HOXB7 in esophageal cancer included in the GTEx and TCGA databases, which also revealed a markedly abundant expression of HOXB7 in esophageal cancer (Figure 1B). Subsequently, the expression of HOXB7 was determined by RT-qPCR, Western blot analysis and IHC in esophageal cancer tissues and adjacent normal tissues. Results of IHC revealed that HOXB7 was primarily localized in the nucleus as brownish-yellow staining, with higher positive expression rate of HOXB7 protein in esophageal cancer tissues compared to adjacent normal tissues (*p* < 0.05, Figures 1C,D). RT-qPCR and Western blot analysis further verified that HOXB7 expression was much higher in esophageal cancer tissues relative to adjacent normal tissues (*p* < 0.05, Figures 1E–G). The aforementioned findings demonstrated that HOXB7 was highly-expressed in esophageal cancer.

### Over-Expression of HOXB7 Enhances Resistance of KYSE150 and KYSE150R Cells to Radiotherapy *in vitro*

**FIGURE 2:**
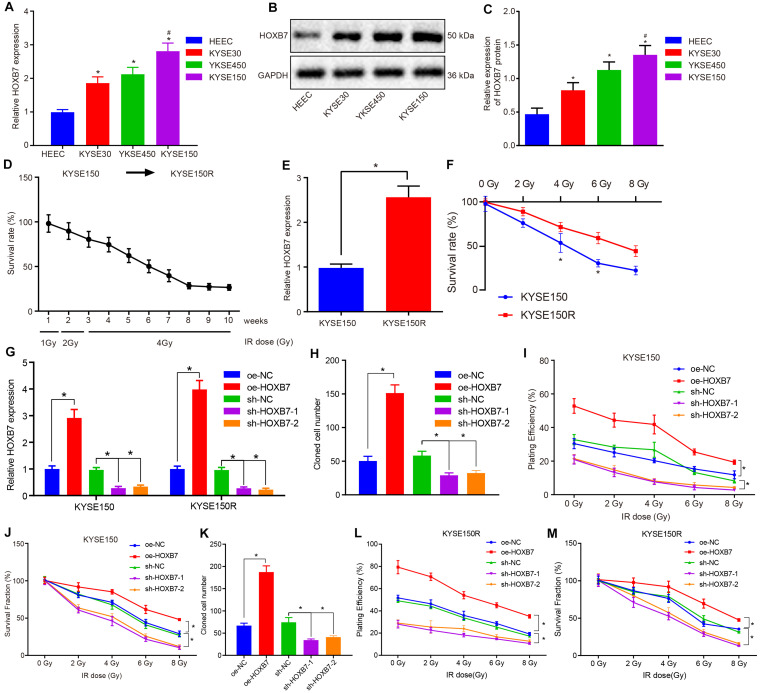
HOXB7 silencing exerts inhibitory effects on resistance of KYSE150 and KYSE150R cells to radiotherapy *in vitro*. **(A)** mRNA expression of HOXB7 determined by RT-qPCR in normal esophageal epithelial cell line (HEEC) and esophageal cancer cell lines (KYSE30, KYSE150, and KYSE450). **(B)** Representative Western blots of HOXB7 protein in normal esophageal epithelial cell line (HEEC) and esophageal cancer cell lines (KYSE30, KYSE150, and KYSE450), normalized to GAPDH. **(C)** Quantitation of panel **(B)**. **(D)** Survival rate of KYSE150 cell lines exposed to different doses of X-ray irradiation. Linear accelerator X-ray irradiation was used to irradiate cells in logarithmic growth phase and good condition, and the irradiation dose was gradually increased in order to screen and verify the survival rate of cell lines. The abscissa represents cell lines irradiated with different doses at different time points, and the ordinate represents the survival coefficient of the corresponding cell lines. After 10 weeks, the original KYSE150 cells were mutated into KYSE150R cell lines. **(E)** mRNA expression of HOXB7 determined by RT-qPCR in KYSE150 and radioresistant cell line KYSE150R. **(F)** Quantitative analysis of the survival rate of KYSE150 and KYSE150R cells in response to radiation at doses ranging from 2 to 8 Gy. KYSE150 and KYSE150R cells were infected with oe-HOXB7 or sh-HOXB7. **(G)** The expression of HOXB7 detected by RT-qPCR in KYSE150 and KYSE150R cells. **(H)** Representative images showing PE of KYSE150 cells detected by colony formation assay. **(I)** Quantitation of panel **(H)**. When the cell clones were visible to the naked eyes, approximately 50 cells in each clone were counted under a microscope, then fixed with 4% paraformaldehyde and stained with crystal violet, followed by PE calculation. **(J)** SF of KYSE150 cells measured by colony formation assay. When the cell clones were visible to the naked eyes, approximately 50 cells in each clone were counted under a microscope, then fixed with 4% paraformaldehyde and stained with crystal violet, followed by SF calculation. **(K)** Representative images showing PE of KYSE150R cells detected by colony formation assay. **(L)** Quantitation of panel **(K)**. **(M)** SF of KYSE150R cells measured by colony formation assay. *p < 0.05 versus HEEC cell lines, KYSE150 cell lines, oe-NC group (KYSE150 or KYSE150R cells treated with oe-NC) or sh-NC group (KYSE150 or KYSE150R cells treated with sh-NC). ^#^*p* < 0.05 versus the KYSE150 cell line. Data were shown as mean ± SD of three technical replicates. Comparisons between two groups were conducted by independent sample *t*-test, while those among multiple groups were conducted by one-way analysis of variance, followed by Tukey’s test. Comparisons at different time points among multiple groups were conducted by two-way analysis of variance, followed by Tukey’s test.

Reverse transcription quantitative polymerase chain reaction was employed to determine the expression of HOXB7 in human esophageal cancer cell lines (KYSE30, KYSE150, and KYSE450) and normal esophageal epithelial cell line (HEEC). The KYSE30, KYSE150, and KYSE450 cell lines presented with significantly high mRNA and protein expression of HOXB7 compared to the HEEC cell lines, among which the KYSE150 cell line exhibited the highest HOXB7 expression (*p* < 0.05, Figures 2A–C), and was therefore selected for subsequent experimentation. Radioresistant cell lines were established using the fractionated irradiation method. After 10 weeks of gradually progressive exposure to 1, 2, or 4 Gy of X-ray irradiation, radioresistant cells in stable passage were obtained and designated as the KYSE150R cells (Figure 2D). We further assessed the expression of HOXB7 in KYSE150 and KYSE150R cells to confirm the correlation between HOXB7 and resistance of esophageal cancer cells to radiotherapy. As shown in Figure 2E, HOXB7 expression was higher in KYSE150R cells than in KYSE150 cells (*p* < 0.05). In addition, Figure 2F reveals that KYSE150R cells exposed to radiation from 2 to 8 Gy exhibited stronger resistance to radiotherapy in contrast to parental KYSE150 cells (*p* < 0.05). Subsequently, the vectors of oe-NC, oe-HOXB7, sh-NC, and sh-HOXB7 were used to infect KYSE150 and KYSE150R cells. RT-qPCR analysis revealed that HOXB7 expression was increased in cells overexerting HOXB7, while it was decreased following sh-HOXB7 treatment (Figure 2G). Additionally, analysis using colony formation assay revealed HOXB7 was over-expressed in KYSE150 or KYSE150R, the colony formation of the cells was found to be enhanced with the increase of PE and SF (*p* < 0.05). Whereas, silencing of HOXB7 brought about a decline in the colony formation (*p* < 0.05) (Figures 2H–M). Taken together, these findings suggested that esophageal cancer cells with HOXB7 silencing were more susceptible to X-ray irradiation and HOXB7 enhanced radioresistance of the cancer cells *in vitro*.

### Over-Expression of HOXB7 Promotes Proliferation and Reduces Apoptosis of KYSE150 and KYSE150R Cells *in vitro*

**FIGURE 3:**
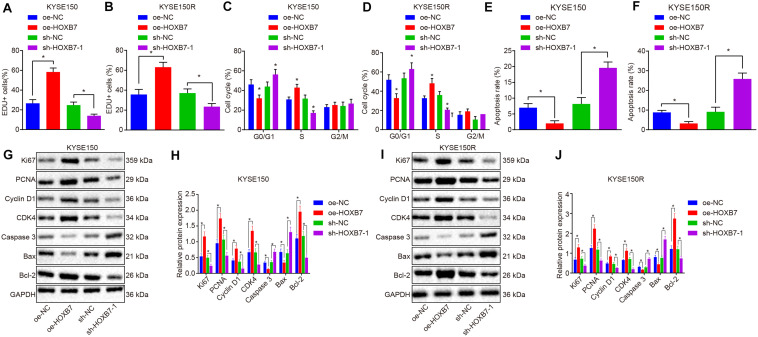
HOXB7 silencing inhibits proliferation and promotes apoptosis of KYSE150 and KYSE150R cells *in vitro*. KYSE150 and KYSE150R cells were infected with oe-HOXB7 or sh-HOXB7. **(A)** Proliferation of KYSE150 cells detected by EdU assay (200×). **(B)** Proliferation of KYSE150R cells detected by EdU assay (200×). The cells in the logarithmic growth phase at 48 h post infection were labeled by EdU. Six to ten fields of view were randomly selected from each well for observation and photograph under a fluorescence microscope, followed by statistical analysis. **(C)** KYSE150 cell cycle distribution measured by flow cytometry. **(D)** KYSE150R cell cycle distribution detected by flow cytometry. After 48 h of infection, the cells were collected and stained with PI. The red fluorescence was then recorded at an excitation wavelength of 488 nm and the cell cycle was detected using flow cytometry. **(E)** KYSE150 cell apoptosis detected by flow cytometry. **(F)** KYSE150R cell apoptosis detected by flow cytometry. **(G)** Representative Western blots of pro-proliferation (Ki67, PCNA, cyclin D1, and CDK4), pro-apoptosis (Bax and caspase 3), and anti-apoptosis (Bcl-2) proteins in KYSE150 cells, normalized to GAPDH. **(H)** Quantitation of panel **(G)**. **(I)** Representative Western blots of pro-proliferation (Ki67, PCNA, cyclin D1, and CDK4), pro-apoptosis (Bax and caspase 3), and anti-apoptosis (Bcl-2) proteins in KYSE150R cells, normalized to GAPDH. **(J)** Quantitation of panel **(I)**. ^∗^*p* < 0.05 versus the oe-NC group (KYSE150 or KYSE150R cells treated with oe-NC) or sh-NC group (KYSE150 or KYSE150R cells treated with sh-NC). Data were shown as mean ± SD of three technical replicates. Comparisons between two groups were conducted by independent sample *t*-test, while those among multiple groups were conducted by one-way analysis of variance, followed by Tukey’s test.

Next, we shifted our attention to examine the effects of HOXB7 on proliferation and apoptosis in KYSE150 and KYSE150R cells. According to the results of 5-ethynyl-2′-deoxyuridine (EdU) assay and flow cytometry, KYSE150 cell proliferation was promoted (Figure 3A), while apoptosis was suppressed (Figure 3E) by delivery of oe-HOXB7, with fewer cells arrested at the G0/G1 phase and more cells arrested at the S and G2/M phases (Figure 3C). All these effects were reversed by delivery of sh-HOXB7 (*p* < 0.05). Consistent results in cell proliferation (Figure 3B), cell cycle entry (Figure 3D), and apoptosis (Figure 3F) were observed in KYSE150R cells. Additionally, the protein expression of pro-proliferation factors (Ki67, PCNA, cyclin D1, and CDK4), anti-apoptosis factor (Bcl-2) and pro-apoptosis factor (Bax and caspase 3) was measured by Western blot analysis. The results demonstrated that the protein expression of Ki67, PCNA, cyclin D1, CDK4 and Bcl-2 was increased, while that of Bax and caspase 3 was decreased in KYSE150 (Figures 3G,H) and KYSE150R cells (Figures 3I,J) over-expressing HOXB7, while silencing HOXB7 brought about opposite results (*p* < 0.05). Hence, it could be inferred that HOXB7 silencing inhibited the proliferative ability of KYSE150 and KYSE150R cells while boosting the apoptotic potential *in vitro*.

### MAGI2-AS3 Is Poorly Expressed in Esophageal Cancer and Negatively Regulates HOXB7 *in vitro*

**FIGURE 4:**
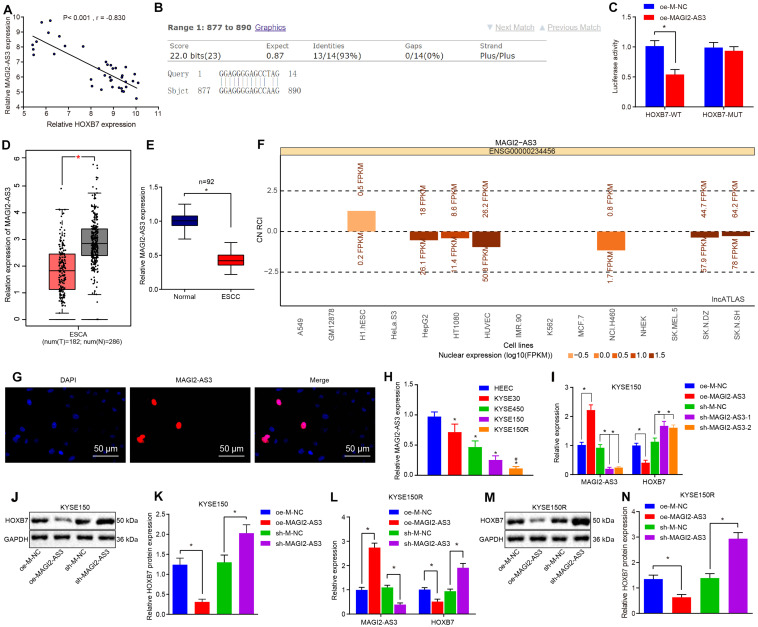
Low expression of MAGI2-AS3 in esophageal cancer. **(A)** Correlation analysis between MAGI2-AS3 expression and HOXB7 expression in the GSE45670 dataset. **(B)** Binding of MAGI2-AS3 to the HOXB7 promoter sequence analyzed by Blast. **(C)** Luciferase activity of HOXB7-WT and HOXB7-MUT determined by dual-luciferase reporter gene assay. **(D)** Expression of MAGI2-AS3 in normal tissues (gray) and esophageal cancer tissues (red) analyzed by the GEPIA database. **(E)** Expression of MAGI2-AS3 determined by RT-qPCR in adjacent normal and esophageal cancer tissues. **(F)** Subcellular localization of MAGI2-AS3 predicted by the lncATLAS database. The column in the figure shows the expression of lncRNA, in which when the column is above line 0, it indicates that the lncRNA is mainly expressed in the cytoplasm and below line 0, it is mainly expressed in the nucleus. **(G)** Subcellular localization of MAGI2-AS3 identified by FISH assay in KYSE150 cells (200×). **(H)** mRNA expression of MAGI2-AS3 determined by RT-qPCR in normal esophageal epithelial cell line (HEEC) and esophageal cancer cell lines (KYSE30, KYSE150, and KYSE450). KYSE150 and KYSE150R cells were infected with oe-MAGI2-AS3 or sh-MAGI2-AS3 **(I)** mRNA expression of HOXB7 and MAGI2-AS3 determined by RT-qPCR in KYSE150 cell lines. **(J)** Representative Western blots of HOXB7 protein in KYSE150 cell lines, normalized to GAPDH. **(K)** Quantitation of panel **(J)**. **(L)** Expression of MAGI2-AS3 and HOXB7 detected by RT-qPCR in KYSE150 cell lines. **(M)** Representative Western blots of HOXB7 protein in KYSE150R cell lines, normalized to GAPDH. **(N)** Quantitation of panel **(M)**. ^∗^*p* < 0.05 versus the adjacent normal tissues, HEEC cell lines, oe-M-NC group (KYSE150 or KYSE150R cells treated with oe-M-NC) or sh-M-NC group (KYSE150 or KYSE150R cells treated with sh-M-NC). ^#^*p* < 0.05 versus the KYSE150 cell line. Data were shown as mean ± SD of three technical replicates. Comparisons between two groups were conducted by independent sample *t*-test while those among multiple groups were conducted by one-way analysis of variance, followed by Tukey’s test.

Studies have shown that MAGI2-AS3 plays an important role in cancer (Wang et al., 2018; Yang et al., 2018). To explore the regulatory mechanism of HOXB7 in esophageal cancer, correlation analysis was conducted in GSE45670 dataset, which revealed a negative correlation between MAGI2-AS3 expression and HOXB7 expression (Figure 4A). MAGI2-AS3 RNA could bind to the DNA sequence of the HOXB7 promoter (Figure 4B). Binding of MAGI2-AS3 to the promoter region of HOXB7 was further confirmed using dual-luciferase reporter gene assay. As depicted in Figure 4C, MAGI2-AS3 could negatively regulate the transcriptional activity of the HOXB7 promoter region (*p* < 0.05), indicating that MAGI2-AS3 could bind to the HOXB7 promoter region, which was in line with the results of the Blast analysis. Furthermore, the GEPIA database^6^ indicated that MAGI2-AS3 was poorly-expressed in esophageal cancer (Figure 4D), and subsequent determination of MAGI2-AS3 levels in esophageal cancer and adjacent normal tissues confirmed that result (Figure 4E). Moreover, MAGI2-AS3 was identified to localize primarily in the nucleus by lncATLAS website (see text footnote 3) and FISH assay (Figures 4F,G). Then, the expression of MAGI2-AS3 was assessed using RT-qPCR in KYSE30, KYSE150, KYSE450, and HEEC cell lines. The results revealed that the expression of MAGI2-AS3 was much lower in KYSE30, KYSE150, and KYSE450 cell lines than that in HEEC cell lines. Among these the KYSE150 cell line presented with the lowest expression of MAGI2-AS3, while MAGI2-AS3 expression was even lower in the KYSE150R cell line relative to the KYSE150 cell line (*p* < 0.05, Figure 4H).

Consequently, to investigate whether MAGI2-AS3 regulates the expression of HOXB7, lentiviral vectors of oe-M-NC, oe-MAGI2-AS3, sh-M-NC, and sh-MAGI2-AS3 were transduced into KYSE150 and KYSE150R cells, respectively. The changes in the expression of MAGI2-AS3 and HOXB7 were analyzed by RT-qPCR, which showed that over-expression of MAGI2-AS3 markedly decreased the expression of HOXB7, while treatment with sh-MAGI2-AS3-1 and sh-MAGI2-AS3-2 downregulated MAGI2-AS3 expression and promoted HOXB7 expression in cells (Figures 4I–N) (*p* < 0.05). The obtained evidence suggested that MAGI2-AS3 was negatively correlated with HOXB7, and MAGI2-AS3 localized in the nucleus was poorly-expressed in esophageal cancer, and down-regulated HOXB7 transcription *in vitro*.

### MAGI2-AS3 Down-Regulates HOXB7 by Recruiting EZH2 to Initiate H3K27me3

**FIGURE 5:**
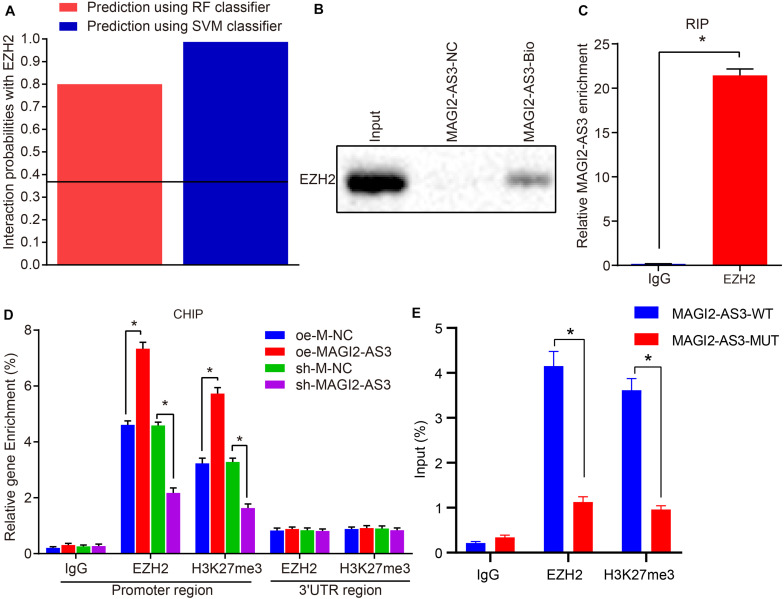
MAGI2-AS3 down-regulates HOXB7 expression by promoting H3K27me3 methylation *via* EZH2. **(A)** Interaction probabilities of MAGI2-AS3 with EZH2 where values of RF and SVM > 0.5 were indicative of binding ability. **(B)** RNA pull-down assay combined with Western blot assay to assess EZH2 enrichment on MAGI2-AS3. **(C)** RIP assay to detect the recruitment of EZH2 by MAGI2-AS3. **(D)** Enrichment of EZH2 and H3K27me3 in the HOXB7 promoter region detected by ChIP assay in cells treated with oe-MAGI2-AS3 or sh-MAGI2-AS3. **(E)** Enrichment of EZH2 and H3K27me3 in the HOXB7 promoter region detected by ChIP assay in the presence of MAGI2-AS3-WT or MAGI2-AS3-MUT. *p < 0.05 versus the IgG group, oe-M-NC group (KYSE150 or KYSE150R cells treated with oe-M-NC) or sh-M-NC group (KYSE150 or KYSE150R cells treated with sh-M-NC). Data were shown as mean ± SD of three technical replicates, and compared by independent sample *t*-test.

As a stemness factor, histone methyltransferase EZH2 has the ability to regulate cell differentiation, embryonic development, and cancer development, and EZH2 silencing has been confirmed to downregulate different genes in ESCC (Karami Madani et al., 2018). H3K27me3 is required for EZH2-mediated repression of various genes essential for tumorigenesis and tumor development, while the expression of H3K27me3 and EZH2 could serve as biomarkers in the prediction of ESCC patients’ survival and ESCC metastasis (Liu et al., 2016). In addition, existing evidence has indicated a correlation between HOXB7 activity and the loss of H3K27me3 (di Pietro et al., 2012). Meanwhile, EZH2 can catalyze H3K27me3 to regulate gene expression through epigenetic mechanisms (Gan et al., 2018). Therefore, we speculated that EZH2 could promote the expression of H3K27me3 and inhibit the activation of HOXB7. Results of the RPIseq database search^7^ predicted that MAGI2-AS3 could bind to EZH2 (Figure 5A). In addition, analysis on the enrichment of MAGI2-AS3 to EZH2 using RNA pull-down and RIP assays found that the enrichment of EZH2 on MAGI2-AS3 was increased in cells overexpressing EZH2 (*p* < 0.05, Figures 5B,C). To investigate further the relation between MAGI2-AS3, EZH2, H3K27me3, and HOXB7 methylation, esophageal cancer cells were infected with vectors of oe-M-NC, oe-MAGI2-AS3, sh-M-NC, and sh-MAGI2-AS3. ChIP assay showed that the enrichment of EZH2 and H3K27me3 in the HOXB7 promoter region was higher in cells over-expressing MAGI2-AS3, while opposite findings were observed in cell lines with MAGI2-AS3 knockdown (*p* < 0.05, Figure 5D). Meanwhile, EZH2 and H3K27me3 showed lower enrichment in the HOXB7 promoter region in the presence of MAGI2-AS3-MUT than MAGI2-AS3-WT (Figure 5E). These findings confirmed that MAGI2-AS3 could promote the enrichment of EZH2 and H3K27me3 in HOXB7 promoter region. The aforementioned findings highlighted that MAGI2-AS3 recruited EZH2 to the HOXB7 promoter to initiate H3K27me3 and suppress the expression of HOXB7.

### MAGI2-AS3 Silencing Strengthens Resistance of KYSE150R Cells to Radiotherapy *in vivo*

**FIGURE 6:**
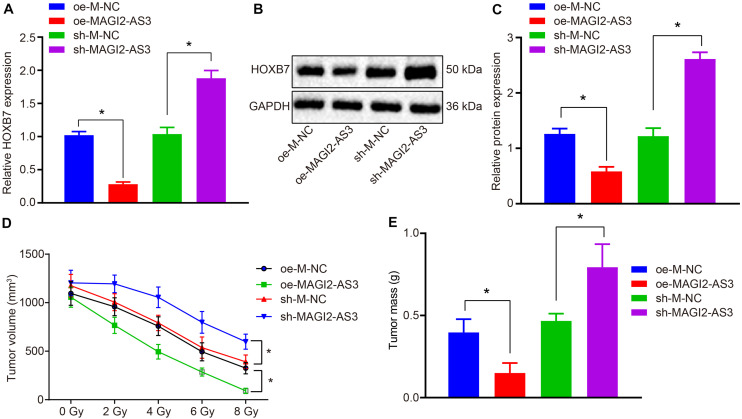
MAGI2-AS3 silencing promotes KYSE150R cell resistance to radiotherapy *in vivo*. Nude mice were injected with KYSE150R cells infected with lentivirus expressing oe-MAGI2-AS3 or sh-MAGI2-AS3. **(A)** mRNA expression of HOXB7 determined by RT-qPCR in tumor tissues. **(B)** Representative Western blots of HOXB7 protein normalized to GAPDH in tumor tissues. **(C)** Quantitation of panel **(B)**. **(D)** The growth rate of human esophageal cancer xenograft tumors in nude mice. **(E)** Quantitative analysis of tumor weight. *p < 0.05 versus the oe-M-NC group (nude mice inoculated with KYSE150R cells infected by oe-M-NC) or sh-M-NC group (nude mice inoculated with KYSE150R cells infected by sh-M-NC). Data were shown as mean ± SD of three technical replicates, and compared by independent sample *t*-test.

To investigate the effect of MAGI2-AS3 on the resistance of esophageal cancer cells to radiotherapy *in vivo*, KYSE150R cells infected with lentivirus expressing oe-M-NC, oe-MAGI2-AS3, sh-M-NC, or sh-MAGI2-AS3 were inoculated into the nude mice. The mice were subjected to X-ray exposure after formation of xenograft tumors. Following euthanasia, the tumor tissues were excised, and their expression of HOXB7 was determined by RT-qPCR, which revealed that HOXB7 was inhibited in tumor tissues of mice treated with oe-MAGI2-AS3, while it was promoted following treatment with sh-MAGI2-AS3 (*p* < 0.05, Figure 6A). Additionally, Western blot analysis of HOXB7 protein revealed that over-expression of MAGI2-AS3 suppressed its expression and conversely, MAGI2-AS3 silencing enhanced the expression in mouse tumor tissues (*p* < 0.05, Figures 6B,C). According to tumor growth curve and weight analyses, tumor growth was slowed down and tumor volume was smaller after X-ray exposure to 0, 2, 4, 6, and 8 Gy. Moreover, tumor size was much smaller (Figure 6D), tumor weight was much lighter (Figure 6E) in mice after inoculation of cells with over-expressed MAGI2-AS3 than after inoculation of cells treated with oe-M-NC (*p* < 0.05). Inoculation of cells in the presence of sh-MAGI2-AS3 was found to lead to opposite results with respect to tumor growth. To conclude, MAGI2-AS3 silencing promoted the resistance of esophageal cancer cells to radiotherapy *in vivo*.

### MAGI2-AS3 Inhibits KYSE150 and KYSE150R Cell Proliferation and Resistance to Radiotherapy While Promoting Cell Apoptosis by Down-Regulating HOXB7 *in vitro*

**FIGURE 7:**
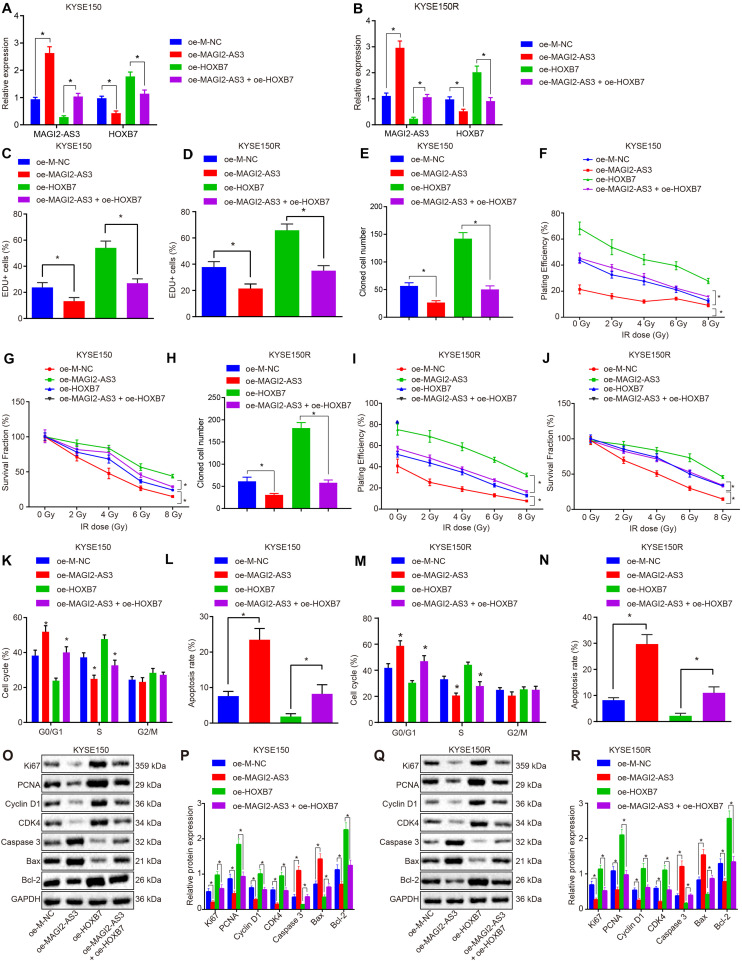
MAGI2-AS3 over-expression inhibits KYSE150 and KYSE150R cell proliferation and radio-resistance through down-regulation of HOXB7 *in vitro*. **(A)** Expression of HOXB7 determined by RT-qPCR in KYSE150 cells. **(B)** Expression of HOXB7 determined by RT-qPCR in KYSE150R cells. KYSE150 and KYSE150R cells were infected with lentivirus expressing oe-MAGI2-AS3, oe-HOXB7, or both. **(C)** KYSE150 cell proliferation detected by EdU assay (200×). **(D)** KYSE150R cell proliferation detected by EdU assay (200×). **(E)** KYSE150 cell colony formation measured by colony formation assay. **(F)** SF of KYSE150 cells measured by colony formation assay. **(G)** PE of KYSE150 cells measured by colony formation assay. **(H)** KYSE150R cell colony formation measured by colony formation assay. **(I)** SF of KYSE150R cells measured by colony formation assay. **(J)** PE of KYSE150R cells measured by colony formation assay. **(K)** KYSE150 cell cycle distribution detected by flow cytometry. **(L)** KYSE150 cell apoptosis detected by flow cytometry, and PI and Annexin-V-FITC double positive cells are apoptotic cells. **(M)** KYSE150R cell cycle distribution detected by flow cytometry. **(N)** KYSE150R cell apoptosis detected by flow cytometry, and PI and Annexin-V-FITC double positive cells are apoptotic cells. **(O)** Representative Western blots of pro-proliferation (Ki67, PCNA, cyclin D1, and CDK4), pro-apoptosis (Bax), and anti-apoptosis (Bcl-2) proteins normalized to GAPDH in KYSE150 cells. **(P)** Quantitation of panel **(O)**. **(Q)** Representative Western blots of pro-proliferation (Ki67, PCNA, cyclin D1, and CDK4), pro-apoptosis (Bax and caspase 3), and anti-apoptosis (Bcl-2) proteins normalized to GAPDH in KYSE150R cells. **(R)** Quantitation of panel **(Q)**. *p < 0.05 versus the oe-M-NC group (KYSE150 or KYSE150R cells infected by oe-M-NC) or oe-HOXB7 group (KYSE150 or KYSE150R cells infected by oe-HOXB7). Data are shown as mean ± SD of three technical replicates. Comparisons between two groups were conducted by independent sample *t*-test while those among multiple groups were conducted by one-way analysis of variance, followed by Tukey’s test. Comparisons at different time points among multiple groups were conducted by two-way analysis of variance, followed by Tukey’s test.

Having identified that MAGI2-AS3 regulated radio-resistance of esophageal cancer cells *in vivo*, we focused our attention on the effects of HOXB7 on esophageal cancer cell proliferation, apoptosis, and resistance to radiotherapy. KYSE150 and KYSE150R cells were, respectively, infected with lentivirus expressing oe-M-NC, oe-MAGI2-AS3, oe-HOXB7, or oe-MAGI2-AS3 + oe-HOXB7. RT-qPCR detected that the expression of MAGI2-AS3 was elevated while that of HOXB7 was inhibited upon over-expression of MAGI2-AS3 in cells (*p* < 0.05). Treatment with oe-HOXB7 increased the expression of HOXB7, whereas dual treatment with oe-MAGI2-AS3 and oe-HOXB7 upregulated MAGI2-AS3 expression but decreased HOXB7 expression in cells (*p* < 0.05, Figures 7A,B). Changes in the expression of HOXB7 also affected the proliferation, apoptosis and radio-resistance of cancer cells. Alternations with regard to cellular biological functions were further explored through EdU assay, colony formation survival assay and flow cytometry. Results indicated that restoring MAGI2-AS3 suppressed the proliferation of KYSE150 and KYSE150R cells (Figures 7C,D) and resistance to radiotherapy (Figures 7E–J), decreased PE and SF and promoted apoptosis (Figures 7M,N) with more cells arrested at the G0/G1 phase and fewer cells arrested at the S and G2/M phases (*p* < 0.05) (Figures 7K,L). However, opposite results were detected in KYSE150 and KYSE150R cells treated with restored HOXB7 (*p* < 0.05). Comparisons between cells in the presence of oe-HOXB7 and cells in the presence of oe-MAGI2-AS3 + oe-HOXB7 revealed that more highly significant changes were observed in cells treated with both oe-MAGI2-AS3 and oe-HOXB7 (*p* < 0.05). Subsequently, the protein expression of pro-proliferation factors (Ki67, PCNA, cyclin D1, and CDK4), pro-apoptosis factor (Bax and caspase 3) and anti-apoptosis factor (Bcl-2) were measured using Western blot analysis for verification. Consistently, the expressions of Ki67, PCNA, Bcl-2, cyclin D1, and CDK4 were all decreased, while those of Bax and caspase 3 were increased in KYSE150 and KYSE150R cells infected with oe-MAGI2-AS3, whereas KYSE150 and KYSE150R cells infected with oe-HOXB7 presented with opposite results (*p* < 0.05) (Figures 7O–R). Moreover, the results were more pronounced in cells infected with both oe-MAGI2-AS3 and oe-HOXB7 than oe-HOXB7 alone (*p* < 0.05). To sum up, MAGI2-AS3 restoration impeded esophageal cancer cell radio-resistance through inhibition of HOXB7 *in vitro*.

### MAGI2-AS3 Suppresses KYSE150R Cell Resistance to Radiotherapy *in vivo* by Down-Regulating HOXB7

**FIGURE 8:**
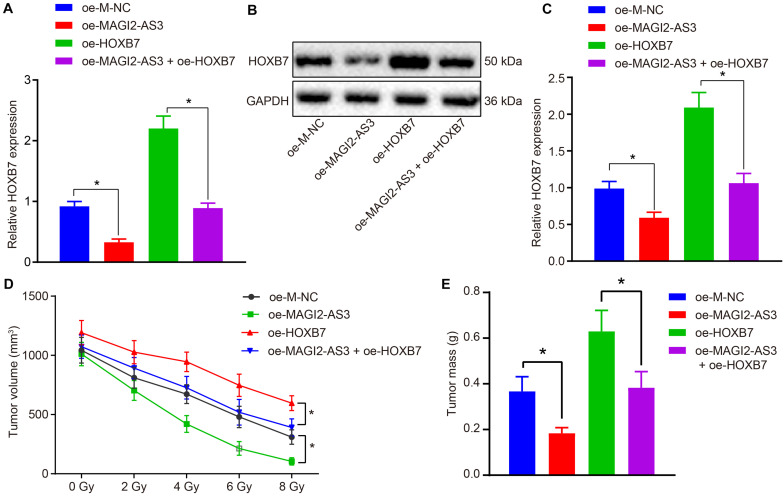
Over-expression of MAGI2-AS3 reduces radio-resistance of KYSE150R cells *in vivo* by negatively regulating HOXB7. Nude mice were injected with KYSE150R cells infected with lentivirus expressing oe-MAGI2-AS3, oe-HOXB7, or both. **(A)** mRNA expression of HOXB7 determined by RT-qPCR in tumor tissues. **(B)** Representative Western blots of HOXB7 protein normalized to GAPDH in tumor tissues. **(C)** Quantitation of panel **(B)**. **(D)** The growth of human esophageal cancer xenograft tumor in nude mice. **(E)** Quantitative analysis of tumor weight. *p < 0.05 versus the oe-M-NC group (nude mice inoculated with KYSE150R cells infected by oe-M-NC) or oe-HOXB7 group (nude mice inoculated with KYSE150R cells infected by oe-HOXB7). Data are shown as mean ± SD of three technical replicates. Comparisons between two groups were conducted by independent sample *t*-test, while those at different time points among multiple groups were conducted by repeated measures analysis of variance, followed by Tukey’s test.

Finally, we investigated whether the role of MAGI2-AS3 in esophageal cancer cells was achieved by down-regulating HOXB7 *in vivo*. KYSE150R cells infected with lentivirus carrying oe-M-NC, oe-MAGI2-AS3, oe-HOXB7 or oe-MAGI2-AS3 + oe-HOXB7 were inoculated into the nude mice. After tumor formation, the mice were further treated with radiotherapy for 1 week. After euthanasia, the tumor tissues were excised, and their expression of HOXB7 was determined by RT-qPCR. As depicted in Figures 8A–C, MAGI2-AS3 over-expression caused a decline in the mRNA and protein expression of HOXB7, while HOXB7 over-expression induced opposite results (*p* < 0.05). In comparison to oe-HOXB7 treatment alone, over-expression of both MAGI2-AS3 and HOXB7 negated the effects of over-expressing HOXB7 alone considering the decreased mRNA and protein expression of HOXB7 (*p* < 0.05). In agreement with our initial assumption, irradiation led to a more pronounced shrinkage of the tumor, as evidenced by reduced tumor volume (Figure 8D) and tumor weight (Figure 8E) in mice injected with KYSE150R cells over-expressing MAGI2-AS3, while mice injected with KYSE150R cells over-expressing HOXB7 presented with opposite trends (*p* < 0.05). As expected, KYSE150R cells over-expressing both MAGI2-AS3 and HOXB7 counteracted the effects of over-expressing HOXB7 alone (*p* < 0.05). Therefore, MAGI2-AS3 restoration could suppress radio-resistance of esophageal cancer cells *in vivo* by down-regulating HOXB7.

## Discussion

Esophageal cancer is one of the leading causes of cancer-related deaths across the world, and is traditionally treated by surgery, radiotherapy, and chemotherapy (Cools-Lartigue et al., 2015). Among these treatment modalities, radiotherapy is regarded as a safer non-surgical treatment regimen for patients suffering from esophageal cancer (Pottgen and Stuschke, 2012). Unfortunately, patients often present with poor clinical outcomes, with the eventual dominance of cell subpopulations exhibiting resistance to radiotherapy and harboring a more aggressive phenotype than the original parental cells (Park et al., 2019). Nevertheless, accumulating evidence has highlighted the function of lncRNAs in mediating the resistance of esophageal cancer cells to chemoradiotherapy (Tong et al., 2014; Li et al., 2017). In the current study, we identified a novel lncRNA, MAGI2-AS3, and explored its specific role in esophageal cancer cell resistance to radiotherapy. Our findings revealed that restoration of MAGI2-AS3 could function as a tumor suppressor by down-regulating its target gene HOXB7, thus attenuating radio-resistance in esophageal cancer cells.

Our initial results demonstrated that esophageal cancer tissues and cell lines exhibited low expression of MAGI2-AS3 and high expression of HOXB7. Similarly, poor expression of MAGI2-AS3 has been detected in breast cancer tissues when compared with adjacent normal tissues and breast cancer cell growth tends to be suppressed in response to MAGI2-AS3 over-expression (Yang et al., 2018). In addition, bladder cancer tissues also exhibit reduced expression of MAGI2-AS3, while restoring MAGI2-AS3 sufficiently impeded the progression of bladder cancer (Wang et al., 2018). Consistently, we infected KYSE150 cells with lentiviral vectors containing over-expressed MAGI2-AS3, which were subcutaneously inoculated into nude mice. Subsequent findings confirmed that introduction of over-expressed MAGI2-AS3 significantly reduced the oncogenic properties of esophageal cancer cells. Moreover, ESCC tissues also exhibit up-regulation of HOXB7, while low HOXB7 expression is associated with favorable survival conditions (Xie et al., 2013). Li et al. (2015) have highlighted the capability of HOXB7 silencing in suppressing tumorigenicity f in ESCC as well. Our findings further illustrated that HOXB7 knockdown led to suppression of KYSE150 cancer cell growth *in vitro* and tumor growth in nude mice. We next established a radioresistant cell line (KYSE150R) from the KYSE150 parental cell line by subjecting the cells to a gradient cumulative irradiation dose. Radiotherapy affects cell proliferation, altering the cell cycle distribution and cell apoptosis (Wang et al., 2014). Hereby, we explored the KYSE150R cell proliferation, cycle distribution and apoptosis. Cellular biological functions KYSE150R cells proved to be consistent with those in the KYSE150 cells, supporting the validation on the effects of MAGI2-AS3 restoration or HOXB7 silencing on radio-resistance of esophageal cancer cells.

Additionally, in the current study we made great efforts to elucidate the underlying regulatory mechanism involving MAGI2-AS3 and HOXB7. MAGI2-AS3 is known to function as an miRNA sponge to inhibit cancer progression. For instance, MAGI2-AS3 has been previously highlighted to serve as an endogenous sponge of miR-374b-5p by directly binding to it, consequently inhibiting hepatocellular carcinoma cell proliferation and migration *in vitro*, while impeding tumor growth *in vivo* (Yin et al., 2019). In addition, it is known that MAGI2-AS3 up-regulates the expression of CCDC19 by sponging miR-15b-5p, and thus suppresses bladder cancer progression (Wang et al., 2018). The current study further revealed that MAGI2-AS3 can down-regulate HOXB7 expression by recruiting EZH2 to initiate the upregulation of H3K27me3 protein expression. As a stemness factor, EZH2 possesses the ability to regulate cell differentiation, embryonic development and cancer development, such that EZH2 silencing demonstrably down-regulates various genes in ESCC (Karami Madani et al., 2018). The indispensable role of H3K27me3 in EZH2-mediated repression of genes in tumorigenesis and tumor progression has also been documented, while expression of H3K27me3 and EZH2 are indicative of poor prognosis of patients with ESCC (Liu et al., 2016). Furthermore, HOXB gene activation has been demonstrated to occur in parallel with H3K27me3 loss also in Barrett’s esophagus-associated adenocarcinoma (di Pietro et al., 2012). Likewise, a similar interplay has been reported in ESCC whereby lncRNA metastasis-associated lung adenocarcinoma transcript 1 can promote malignant development by targeting β-catenin *via* EZH2 and lncRNA cancer susceptibility candidate 9 to enhance ESCC growth by targeting programmed cell death protein 4 *via* EZH2 (di Pietro et al., 2012; Wu et al., 2017).

To sum up, the current study highlights the regulatory mechanism by which MAGI2-AS3 mediates esophageal cancer radio-resistance in terms of cell proliferation and apoptosis *in vitro* and *in vivo*. Furthermore, we show that MAGI2-AS3 and HOXB7 can serve as biomarkers for radio-resistance and that gene therapy through MAGI2-AS3 restoration or HOXB7 silencing may improve the treatment of esophageal cancer by overcoming radioresistance. Nonetheless, whether overexpression of HOXB7 is sufficient to reverse the tumor inhibitory effect of MAGI2-AS3 overexpression remains to be ascertained.

## Data Availability Statement

All datasets presented in this study are included in the article/supplementary material.

## Ethics Statement

The study was conducted under the approval of the Institutional Review Board of Fujian Medical University Cancer Hospital, Fujian Cancer Hospital. Signed informed consents were obtained from all participants prior to enrollment. All animal experiments were performed in accordance with the Guide for the Care and Use of Laboratory animals published by the United States National Institutes of Health.

## Author Contributions

WC, XS, and ML designed the study. QY, JM, and JL collated the data, carried out data analyses, and produced the initial draft of the manuscript. WC, XS, and JL contributed to drafting the manuscript. All authors have read and approved the final submitted manuscript.

## Conflict of Interest

The authors declare that the research was conducted in the absence of any commercial or financial relationships that could be construed as a potential conflict of interest.

## Footnotes

1 https://www.ncbi.nlm.nih.gov/geo/

2 http://gepia.cancer-pku.cn/

3 http://lncatlas.crg.eu/

4 https://blast.ncbi.nlm.nih.gov/Blast.cgi?PAGE=MegaBlast&PROGRAM=blastn&BLAST_PROGRAMS=megaBlast&PAGE_TYPE=BlastSearch&BLAST_SPEC=blast2seq&DATABASE=n/a

5 http://gepia2.cancer-pku.cn/#analysis

6 http://gepia.cancer-pku.cn/index.html

7 http://pridb.gdcb.iastate.edu/RPISeq/

## Abbreviations

Bax: Bcl-2-associated X protein
BCA: bicinchoninic acid
Bcl-2: B-cell lymphoma 2
BSA: bovine serum albumin
cDNA: complementary DNA
ChIP: chromatin immunoprecipitation
DAB: diaminobenzidine tetrahydrochloride
DAPI: 4′,6-diamidino-2-phenylindole
DEG: differentially expressed gene
EdU: 5-ethynyl-2′-deoxyuridine
ESCC: esophageal squamous cell carcinoma
EZH2: enhancer of zeste homolog 2
FBS: fetal bovine serum
FISH: fluorescence *in situ* hybridization
FITC: fluorescein isothiocyanate
GAPDH: glyceraldehyde-3-phosphate dehydrogenase
GEO: Gene Expression Omnibus
GEPIA: Gene Expression Profiling Interactive Analysis
GTEx: Genotype-Tissue Expression
H3K27me3: trimethylated lysine-27 of histone H3
HEPES: 2-[4-(2-Hydroxyethyl)-1-piperazinyl]ethanesulfonic acid
HOXB7: homeobox protein Hox-B7
IgG: immunoglobulin G
IHC: immunohistochemistry
lncRNAs: long non-coding RNAs
MUT: mutant
NC: negative control
oe: overexpression
PBS: phosphate buffer saline
PBST: phosphate buffered saline with Tween-20
PCNA: proliferating cell nuclear antigen
PE: plating efficiency
PI: propidium iodide
RIP: RNA binding protein immunoprecipitation
RIPA: radioimmunoprecipitation assay
RPMI: Roswell Park Memorial Institute
RT-qPCR: reverse transcription quantitative polymerase chain reaction
SF: survival fraction
sh: short hairpin RNA
SP: streptavidin peroxidase
TBST: Tris-buffered saline Tween-20
TCGA: The Cancer Genome Atlas
TNM: tumor-nodes-metastasis
WHO: World Health Organization
WT: wild type.
